# Investigations on the Role of the MicroRNA-338-5p/Wnt Family Member 2B (WNT2B) Axis in Regulating the Pathogenesis of Nasopharyngeal Carcinoma (NPC)

**DOI:** 10.3389/fonc.2021.684462

**Published:** 2021-06-29

**Authors:** Suzhen Wang, Tianning Yang, Zhengxiang He

**Affiliations:** ^1^ Department of Otolaryngology, Wuwei People’s Hospital, Wuwei, China; ^2^ Department of Otolaryngology, The First Hospital of Lanzhou University, Lanzhou, China

**Keywords:** microRNAs, nasopharyngeal carcinoma, microRNA-338-5p, malignant phenotypes, Wnt family member 2B

## Abstract

**Background:**

The involvement of microRNA-338-5p in modulating NPC pathogenesis is still largely unknown, and this study aimed to investigate this issue.

**Methods:**

The expressions of cancer associated genes were determined by Real-Time qPCR and Western Blot, and cell apoptosis was determined by flow cytometer (FCM). CCK-8 assay and colony formation assay were respectively used to determine cell proliferation and colony formation abilities. Transwell assay was used to evaluate cell migration. The expression levels of Ki67 protein in mice tissues were measured by Immunohistochemistry (IHC) assay.

**Results:**

The present study found that microRNA-338-5p suppressed NPC progression by degrading its downstream target, Wnt family member 2B (WNT2B). Specifically, microRNA-338-5p tended to be low-expressed in NPC tissues and cell lines, compared to the non-tumor nasopharyngeal mucosa tissues and normal nasopharyngeal cell line (NP69). Upregulation of microRNA-338-5p inhibited proliferation, mobility, and epithelial-mesenchymal transition (EMT) in NPC cells *in vitro*, while silencing of microRNA-338-5p had opposite effects. Consistently, microRNA-338-5p suppressed tumorigenesis of NPC cells *in vivo*. In addition, microRNA-338-5p targeted WNT2B for degradation and inhibition, and the inhibiting effects of microRNA-338-5p overexpression on NPC development were reversed by upregulating WNT2B.

**Conclusions:**

Taken together, we concluded that microRNA-338-5p targeted WNT2B to hinder NPC development.

## Background

Nasopharyngeal carcinoma (NPC) is a common head and neck malignancy worldwide (1, 2), that is characterized by highly malignant local invasion and distant metastasis (3, 4), and imposes a huge health burden on human beings. Unfortunately, clinical data indicated that the traditional therapies, such as radiotherapy and chemoresistance, were not efficacious to cure NPC as the result of radio-resistance (5, 6) and chemo-resistance (7, 8), resulting in worse prognosis in NPC patients. Therefore, development of alternative therapy strategies for NPC became necessary and meaningful (9, 10), and uncovering the underlying mechanisms of NPC pathogenesis might be the first step. Recently, researchers focused on screening out tumor suppressors and oncogenes in NPC, and those cancer associated genes included the genes with or without coding abilities (11–14). Especially, the non-coding RNAs always participated in the regulation of cellular functions *via* serving as post-transcriptional regulators, and our team concentrated on delving into the regulating mechanisms of non-coding RNAs, such as circular RNAs (circ-RNAs) (15, 16), long non-coding RNAs (LncRNAs) (13, 14), and microRNAs (miRNAs) (17, 18), in NPC.

Among all the non-coding RNAs, multiple miRNAs had been identified to regulate NPC development in previous publications (17, 18). For example, miR-99a inhibited cell proliferation and served as a prognostic factor in NPC (19), while other researchers noticed that miR-155 acted as an oncogene to facilitate NPC development (20). Interestingly, the role of microRNA-338-5p in regulating cancer progression varied according to different cancer types (21, 22). On the one hand, microRNA-338-5p hampered the development of esophageal squamous cancer (22), on the other, upregulation of microRNA-338-5p promoted cancer metastasis in colorectal cancer (21). Of note, Ying Shan et al. noticed that microRNA-338-5p inhibited migration and proliferation of NPC cells (23), however, the detailed molecular mechanisms still need to be elucidated. According to previous publications (21, 22, 24), miRNAs targeted the 3’ untranslated regions (3’UTR) of their downstream target genes, and the existing information suggested that microRNA-338-5p negatively regulated hypoxia-induced factor 1α (HIF-1α) in NPC cells (23). Our preliminary data suggested that there existed potential binding sites between microRNA-338-5p and 3’UTR of Wnt family member 2B (WNT2B), and WNT2B had been identified as an oncogene to promote NPC progression (25, 26). Nevertheless, up until now, no literature reported the regulating mechanisms of microRNA-338-5p and WNT2B in NPC cells.

Thus, we designed this study to investigate the role of the microRNA-338-5p/WNT2B axis in regulating NPC pathogenesis, which not only broadened our knowledge in this field, but provided novel biomarkers for NPC diagnosis and treatment.

## Methods

### Clinical Samples Collection and Preparation

The NPC tissues (N = 25) and non-tumor nasopharyngeal mucosa tissues (N = 20) were collected from 2014 to 2017 in Wuwei People’s Hospital. None of the patients accepted other therapies, including chemotherapy, radiotherapy, and adjuvant therapies, before surgical resection, and the NPC patients were histologically and clinically judged by two experienced pathologists, and the clinical tissues were frozen at −70°C conditions. The written informed consent forms had been obtained from all the participants, and our clinic-associated experiments were all allowed by the Ethics Committee in Wuwei People’s Hospital, and the approval number was No. 2017DS632913.

### Cell Culture and Vectors Delivery

We purchased the NPC cell line SUNE1 and SUNE2, and normal nasopharyngeal cell line NP69 from the Cell Bank of the Chinese Academy of Sciences (China) and American Type Culture Collection (ATCC, USA). As previously documented (27), the cells were cultured in Dulbecco’s Modified Eagle’s medium (Gibco, USA) containing 10% fetal bovine serum (FBS, Gibco, USA) under the culture conditions at 37°C with 5% CO_2_ humidified atmosphere. The microRNA-338-5p mimic (50 nM) and inhibitor (100 nM) and WNT2B overexpression vectors (100 nM) were designed and synthesized by a commercial third-party company (GenePharma, Shanghai, China), which were all transfected into the cells by using the Lipofectamine 2000 reagent (Invitrogen, USA).

### Real-Time qPCR

A commercial Trizol kit (Invitrogen, USA) was used for RNA extraction in NPC cells and tissues, and Real-Time qPCR was conducted to quantify microRNA-338-5p and WNT2B mRNA, which were respectively normalized by U6 and β-actin. The detailed experimental procedures could be found in the previous publication (28), and the primer sequences had been documented in **Table 1**.

**Table 1:** The primer sequences for Real-Time qPCR.

Gene name	Primer sequences (strand)
microRNA-338-5p	F: 5’‐ATATCCTGGTGCTGAGTG‐3’
	R: 5’‐GAACATGTCTGCGTATCTC‐3’
WNT2B	F: 5’-CCTGTAGCCAGGGTGAACTG-3’
	R: 5’-CGGGCATCCTTAAGCCTCTT-3’
U6	F: 5’‐CTCGCTTCGGCAGCACA‐3’
	F: 5’‐AACGCTTCACGAATTTGCGT‐3’
β-actin	F: 5’-GTCACCAACTGGGACGACAT-3’
	R: 5’-GCCAGAGGCGTACAGGGATA-3’

### Western Blot Analysis

Protein extraction from NPC cells and tissues were finished by using the RIPA lysis buffer reagent (Beyotime, Shanghai, China), and the protein quality was determined by BCA kit (Beyotime, Shanghai, China). Next, proteins were separated by SDS-PAGE, transferred onto the PVDF membranes (Millipore, USA), and incubated with primary antibodies against WNT2B, N-cadherin, and Vimentin, and subsequently with the secondary antibodies. Finally, an electrochemiluminescence (ECL) system was used for protein bands visualization, which were analyzed by using the Image J software. The antibodies’ information had been recorded in **Table 2**.

**Table 2:** The information for the primary antibodies in Western Blot analysis.

Antibodies	Catalog No.	Working concentrations	Company
WNT2B	Ab178418	1:1,500	Abcam, UK
N-cadherin	Ab76057	1:2,000	Abcam, UK
Vimentin	Ab20346	1:1,000	Abcam, UK
β-actin	Ab6276	1:1,500	Abcam, UK

### Cell-Counting Kit-8 (CCK-8) Assay

A commercial CCK-8 kit (YEASEN Biotechnology, Shanghai, China) was used to examine cell proliferation in NPC cells in keeping with the manufacturer’s protocol. Specifically, NPC cells were cultured for different time points, and were subsequently incubated with CCK-8 reaction solution for 2.5 h, which were fully vortexed, and a microplate reader (ThermoFisher Scientific, USA) was employed to determine the optical density (OD) values with 450 nm wavelength.

### Transwell Assay

The NPC cells were cultured in the serum-free medium of the upper chamber of the Invasion Chamber (BD Bioscience, CA, USA) with Matrigel-coated membranes, and the same volume of the medium with 10% FBS was added in the lower chamber to serve as the chemotactic content. The above cells were cultured at 37°C in the incubator for 24 h, and the cells in the upper surface of the membranes were removed, and cells in the lower chamber were fixed with methanol and stained with 0.1% crystal violet for visualization. The stained cells were counted under an invert light microscope.

### Colony Formation Assay

As previously described (29), the colonies, formation abilities in NPC cells were determined by performing colony formation assay. Specifically, NPC cells were cultured in 96-well plates with 1,000 cells/well at 37°C for 14 days, and were subsequently stained with 0.1% crystal violet (Beyotime Biotechnology, Shanghai, China). Then, a light microscope (ThermoFisher Scientific, USA) was used to count the colonies above 15 cells.

### Flow Cytometry (FCM) for Cell Apoptosis Analysis

The NPC cells were respectively stained with Annexin V-FITC and propidium iodide (PI) at room temperature in darkness, and Flow Cytometer (Partec, Germany) was employed to examine cell apoptosis ratio. The cells double stained with Annexin V-FITC and PI were regarded as late apoptosis, with Annexin V-FITC alone being early apoptosis, and with PI alone being necroptosis.

### Bioinformatics Analysis

The targeting sites microRNA-338-5p and 3’UTR of WNT2B mRNA were predicted by the online starBase software (http://starbase.sysu.edu.cn/), and the association of microRNA-338-5p with neck squamous cell carcinoma (HNSC) was analyzed by using the Pan-cancer analysis (http://starbase.sysu.edu.cn/panCancer.php).

### Dual-Luciferase Reporter Gene System Assay

The binding sites in the 3’UTR of WNT2B mRNA were mutated, and the wild-type and mutant WNT2B sequences were cloned into the luciferase reporter plasmids, and named as Wt-WNT2B and Mut-WNT2B, respectively. The above vectors were co-transfected with miR-NC, microRNA-338-5p mimic, and inhibitor into the NPC cells by using the commercial Lipofectamine 2000 reagent (Invitrogen, USA) according to the manufacturer’s protocol. After 48 h post-transfection, the Dual-luciferase reporter gene system (Promega, USA) was employed to determine both firefly luciferase and renilla luciferase activities, and the Luminometer (Promega, USA) was performed to estimate the relative luciferase activities.

### Establishment of Mice Models

The nude female mice (5 weeks old) were purchased from Lanzhou University, and the mice were fed under specific pathogen-free circumstances. The NPC cells were subcutaneously injected into the right flanks of mice at the density of 2 × 10^6^ cells per mouse, and each group contained at least five mice. The caliper was used to measure tumor volumes every 5 days, and at day 25, mice were anesthetized by intravenously injecting Barbiturate (100 mg/kg), and sacrificed by using the cervical dislocation method. The tumors were obtained for further analysis, and the animal experiments were approved by Wuwei People’s Hospital (No. 2017DS632823).

### Immunohistochemistry (IHC) for Ki67 Protein Examination

The expression patterns, including localization and expression levels, were examined by using the IHC assay in mice tumor tissues. The detailed experimental procedures could be found in previous publications (30–32). Briefly, the mice tumor tissues were fixed by paraffin and embedded by wax, and were spliced into about 4-μm thick sections. Next, the IHC was performed by using a Benchmark XT automated staining machine (Ventana, USA), and the primary antibody against Ki67 (Ventana, USA) was diluted into 1:100 to incubate with the tumor tissues. After that, the sections were incubated with the secondary antibody labeled with horseradish peroxidase (HRP, Ventana, USA), and the 3, 3’ diaminobenzidine (DAB) was used to visualize the Ki-67 positive cells. A light microscope was employed to photograph the images to evaluate the expression status of Ki67 protein in mice tumor tissues, and the cells stained in yellow were regarded as Ki67-positive cells.

### Analysis of the Data

Data were presented as Means ± Standard Deviation (SD) and analyzed by SPSS 18.0 software (IBM, USA) and GraphPad Prism 8 (GraphPad Software, USA). Means from two groups were compared by Student’s t-test, and means in multiple groups were analyzed by one-way analysis of variance (ANOVA). Besides, genes’ correlations were analyzed by Pearson Correlation analysis. *P < 0.05 meant statistical significance.

## Results

### MicroRNA-338-5p Was Aberrantly Downregulated in NPC Tissues and Cells

**Figure 1:**
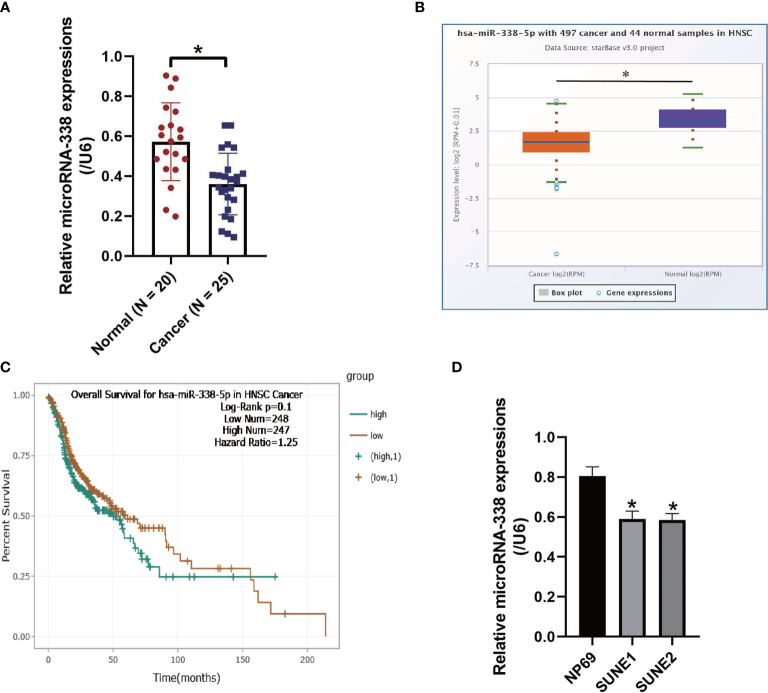
The expression status of microRNA-338-5p were examined in the NPC clinical tissues and cell lines. **(A)** The NPC tissues (N = 25) and non-tumor nasopharyngeal mucosa tissues (N = 20) were obtained, and Real-Time qPCR was conducted to examine the expression levels of microRNA-338-5p. **(B)** The expression levels of microRNA-338-5p in the clinical tissues collected from neck squamous cell carcinoma (HNSC) were analyzed by using the online Pan-cancer analysis. **(C)** Pan-cancer analysis was performed to analyze the correlations of microRNA-338-5p levels and patients’ prognosis in HNSC. **(D)** Real-Time qPCR was used to examine the levels of microRNA-338-5p in NPC cells. Each experiment repeated at least three times, and *P < 0.05.

The NPC tissues (N = 25) and non-tumor nasopharyngeal mucosa tissues (N = 20) were obtained from NPC patients, and Real-Time qPCR was conducted to examine the expression levels of microRNA-338-5p in the above clinical tissues (**Figure 1A**). As shown in **Figure 1A**, we validated that microRNA-338-5p was downregulated in NPC tissues, in contrast with the normal tissues (*P* < 0.05). Consistently, by performing the Pan-cancer analysis, we found that microRNA-338-5p also tended to be low-expressed in the cancer tissues collected from 497 patients with head and neck squamous cell carcinoma (HNSC), instead of the 44 normal samples (*P* < 0.05, **Figure 1B**). In addition, the HNSC patients with low-expressed microRNA-338-5p tended to have a worse prognosis although without statistical significance (*P* = 0.1, **Figure 1C**). Also, the above results were validated by our cellular experiments, which showed that lower levels of microRNA-338-5p were observed in NPC cell lines (SUNE1 and SUNE2), compared to the normal nasopharyngeal cell line (NP69) (*P* < 0.05, **Figure 1D**).

### MicroRNA-338-5p Negatively Regulated Cell Growth and Viability in NPC *In Vitro* and *In Vivo*

**Figure 2:**
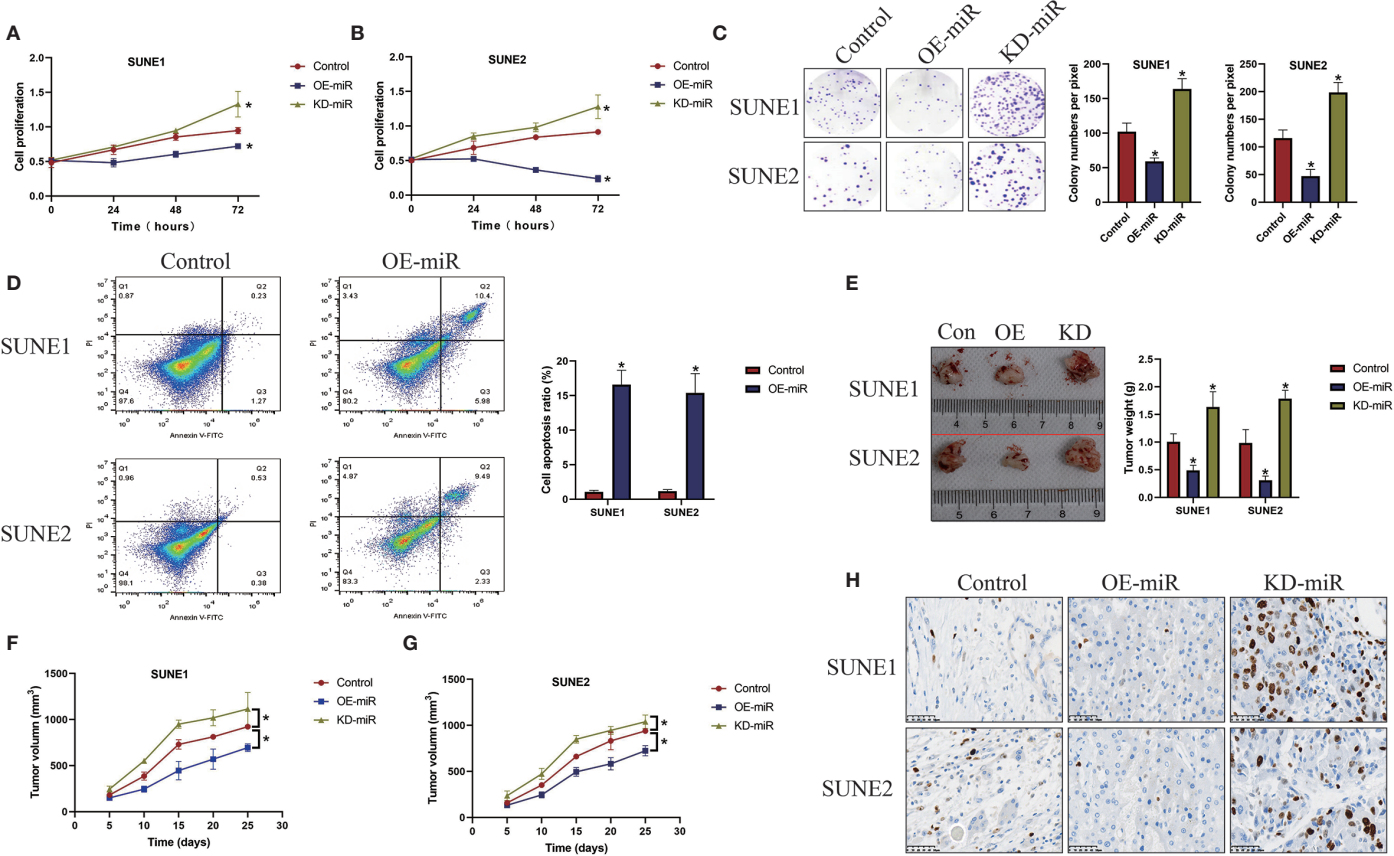
MicroRNA-338-5p inhibited NPC cell proliferation and viability *in vitro* and *in vivo*. **(A, B)** The NPC cells (SUNE1 and SUNE2) were pre-transfected with different vectors, and were cultured for 0, 24, 48, and 72 h, respectively. The CCK-8 assay was performed to evaluate cell proliferation abilities. **(C)** Colony formation assay was used to determine colonies formation abilities in NPC. **(D)** The NPC cells were stained with Annexin V-FITC and PI, and cell apoptosis ratio was measured by flow cytometer (FCM). The xenograft tumor-bearing mice models were established, and **(E)** tumor weight and **(F, G)** volume were measured and documented. **(H)** Immunohistochemistry (IHC) assay was performed to examine the localization and expression levels of Ki67 protein in mice tumor tissues. Each experiment repeated at least three times, and *P < 0.05.

Previous publications reported that the role of microRNA-338-5p in regulating cancer progression is controversial according to cancer types (21, 22), and our data suggested that microRNA-338-5p acted as a tumor suppressor to hinder the development of NPC (**Figure 2**). Specifically, the microRNA-338-5p mimic and inhibitor were constructed and delivered into NPC cells (SUNE1 and SUNE2) to overexpress and downregulate microRNA-338-5p (**Figure S1**), and the CCK-8 assay results showed that microRNA-338-5p negatively regulated cell proliferation abilities in NPC cells (*P* < 0.05, **Figures 2A, B**). Consistently, the colony formation assay evidenced that microRNA-338-5p inhibited colonies formation abilities in NPC cells (*P* < 0.05, **Figure 2C**). Next, by performing the Annexin V-FITC/PI double staining assay, we found that overexpression of microRNA-338-5p triggered apoptotic cell death in NPC cells (*P* < 0.05, **Figure 2D**). In addition, the SUNE1 and SUNE2 cells with differential vectors transfection were used to establish xenograft tumor-bearing mice models, and the results suggested that microRNA-338-5p overexpression slowed down tumor growth (*P* < 0.05, **Figures 2E–G**) and decreased the expression levels of Ki67 protein (**Figure 2H**) to inhibit tumorigenesis of NPC cells *in vivo*, while silencing of microRNA-338-5p had opposite effects (**Figures 2E–H**).

### Upregulation of MicroRNA-338-5p Inhibited NPC Cell Mobility *In Vitro*

**Figure 3:**
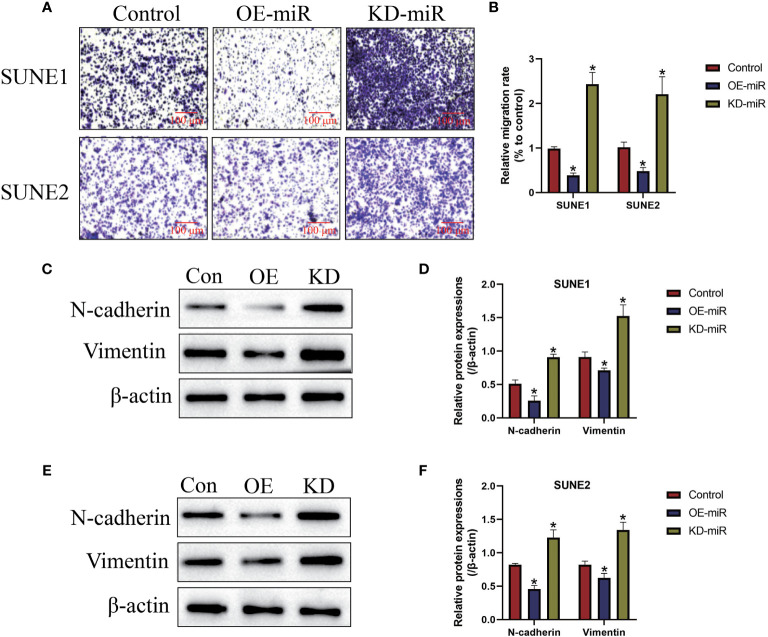
The regulating effects of microRNA-338-5p on cell migration and epithelial-mesenchymal transition (EMT). **(A, B)** Transwell assay was employed to detect cell migration abilities in NPC cells. **(C–F)** Western Blot analysis was conducted to examine the expression levels of EMT associated proteins (N-cadherin and Vimentin) in NPC cells. Each experiment repeated at least three times, and *P < 0.05.

Next, we examined the effects of microRNA-338-5p on cell mobility in NPC cells *in vitro* (**Figure 3**). As shown in **Figures 3A, B**, the transwell assay results showed that upregulation of microRNA-338-5p inhibited cell migration abilities in both SUNE1 and SUNE2 cells (*P* < 0.05), which were significantly inhibited by silencing miR-338-5p (*P* < 0.05). Also, microRNA-338-5p negatively regulated epithelial-mesenchymal transition (EMT) in NPC cells (**Figures 3C–F**). Specifically, the Western Blot analysis results indicated that overexpression of microRNA-338-5p decreased the expression levels of N-cadherin and Vimentin to hamper EMT in NPC cells, and microRNA-338-5p knock-down had opposite effects (*P* < 0.05, **Figures 3C–F**).

### The Regulating Mechanisms of MicroRNA-338-5p and WNT2B in NPC Cells

**Figure 4:**
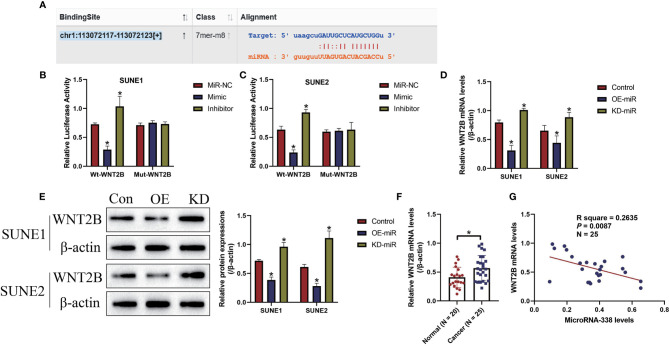
MicroRNA-338-5p targeted 3’ untranslated region (3’UTR) of WNT2B mRNA for its degradation and inhibition. **(A)** The binding sites in microRNA-338-5p and 3’UTR of WNT2B mRNA were predicted by using the online starBase software (http://starbase.sysu.edu.cn/). **(B, C)** The above targeting sites were validated by the dual-luciferase reporter gene system assay in NPC cells. MicroRNA-338-5p negatively regulated the expression levels of WNT2B at both **(D)** transcriptional and **(E)** translated levels, determined by Real-Time qPCR and Western Blot analysis, respectively. **(F)** WNT2B mRNA was upregulated in NPC tissues, compared to the normal tissues. **(G)** Pearson correlation analysis was performed to analyze the correlations between microRNA-338-5p and WNT2B mRNA in NPC tissues. Each experiment repeated at least three times, and *P < 0.05.

The online starBase software (http://starbase.sysu.edu.cn/) predicted that miR-338-5p potentially bound to the 3’ UTR of WNT2B mRNA (**Figure 4A**), and previous data suggested that microRNAs served as post-transcriptional regulators for their downstream target genes. Therefore, we conjectured that there might exist potential regulating mechanisms between miR-338-5p and WNT2B in NPC cells. To investigate this issue, the targeting sites in WNT2B were mutated, and were co-transfected with miR-338-5p mimic and inhibitor into SUNE1 and SUNE2 cells, respectively. The dual-luciferase reporter gene system results indicated that miR-338-5p overexpression decreased the luciferase activity in NPC cells co-transfected with wild-type WNT2B (wt-WNT2B), instead of the mutant WNT2B (Mut-WNT2B), while silencing of miR-338-5p had opposite effects (*P* < 0.05, **Figures 4B, C**). Next, by performing Real-Time qPCR (**Figure 4D**) and Western Blot analysis (**Figure 4E**), we proved that miR-338-5p negatively regulated WNT2B expressions in NPC cells at both transcriptional and translated levels. Also, WNT2B mRNA was upregulated in NPC clinical tissues, in contrast with the normal tissues (*P* < 0.05, **Figure 4F**). Interestingly, miR-338-5p negatively correlated with WNT2B mRNA in NPC tissues (*P* < 0.05, **Figure 4G**).

### Overexpression of MicroRNA-338-5p Hindered NPC Progression by Targeting WNT2B

**Figure 5:**
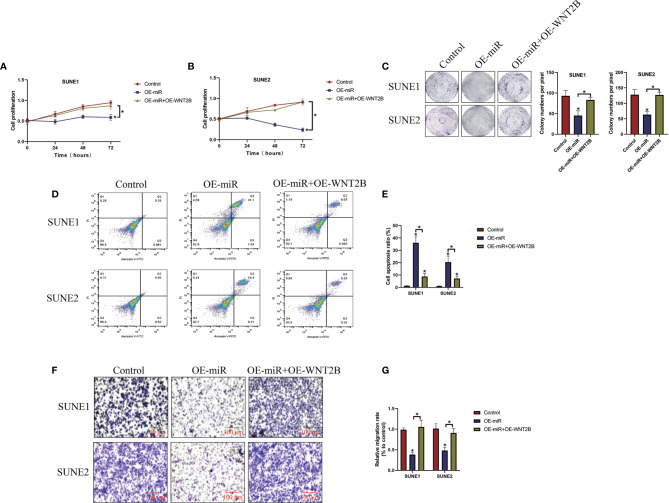
Overexpression of microRNA-338-5p inhibited cancer progression in NPC by inhibiting WNT2B. The NPC cells were transfected with microRNA-338-5p mimic and WNTB overexpression vectors, respectively. **(A, B)** CCK-8 assay was used to examine cell proliferation abilities. **(C)** Colony formation assay was used to determine colonies formation abilities in NPC cells. **(D, E)** Annexin V-FITC/PI double staining assay was performed to measure cell apoptosis ratio. **(F, G)** Transwell assay was performed to examine cell migration abilities. Each experiment repeated at least three times, and *P < 0.05.

Given that miR-338-5p inhibited WNT2B expressions in NPC cells, we next investigated whether miR-338-5p inhibited NPC development by targeting WNT2B. To achieve this, the miR-NC, microRNA-338-5p mimic (OE-miR), and WNT2B overexpression vectors (OE-WNT2B) were delivered into NPC cells, respectively, and the results in **Figure S2** suggested that the OE-WNT2B vectors had been successfully delivered into NPC cells (*P* < 0.05). As expected, the CCK-8 assay (**Figures 5A, B**) and colony formation assay (**Figure 5C**) results showed that WNT2B overexpression abrogated the inhibiting effects of upregulated microRNA-338-5p on both cell proliferation and colonies, formation abilities (*P* < 0.05). Consistently, as shown in **Figures 5D, E**, the Annexin V-FITC/PI double staining assay results showed that overexpression of microRNA-338-5p induced cell apoptosis in NPC cells, which were reversed by upregulating WNT2B (*P* < 0.05). Furthermore, the transwell assay was performed to evaluate cell migration, and we validated that microRNA-338-5p inhibited NPC cell migration through targeting WNT2B (*P* < 0.05, **Figures 5F, G**).

## Discussion

Emerging evidence suggested that miRNAs played important roles in regulating NPC pathogenesis, and targeting the cancer associated miRNAs had been proven as novel strategies to hinder the development of NPC (17, 18). Among all the miRNAs, microRNA-338-5p was identified as a tumor suppressor in colorectal cancer (21), but it served as an oncogene to promote esophageal squamous cancer progression (22), suggesting that the role of microRNA-338-5p in regulating cancer development was controversial based on different cancer types. According to the data from Ying Shan et al., microRNA-338-5p inhibited migration and proliferation of NPC cells (23), but the detailed mechanisms were still not fully delineated. In line with the previous work (23), we validated that microRNA-338-5p functioned as a tumor suppressor to hamper the development of NPC *in vitro* and *in vivo* by targeting its downstream target Wnt family member 2B (WNT2B). Mechanistically, overexpression of microRNA-338-5p inhibited cell proliferation, colonies, formation abilities, migration, epithelial-mesenchymal transition (EMT), and tumorigenesis, while promoted cell apoptosis in NPC cells. Consistently, knock-down of microRNA-338-5p had opposite effects on the above malignant phenotypes.

Based on the existing information from the previous publications (21, 22, 24), miRNAs often targeted the 3’ untranslated regions (3’UTRs) of their downstream target genes for degradation and inhibition, to regulate biological functions in cancer cells. In addition, multiple cancer associated genes, such as hypoxia-induced factor 1α (HIF-1α) (23), Sox 4 (33), E26 transformation specific-1 (ETS1) (34), and sphingosine kinase 2 (SphK2) (35), could be targeted by microRNA-338-5p. Therefore, we conjectured that microRNA-338-5p might regulate NPC progression by inhibiting its corresponding downstream cancer associated genes in a similar manner. To validate this hypothesis, the online starBase software (http://starbase.sysu.edu.cn/) was used, and we predicted that microRNA-338-5p potentially bound to the 3’ UTR of WNT2B mRNA. Furthermore, we validated that microRNA-338-5p negatively regulated WNT2B expressions in NPC cells at both transcriptional and translated levels. Interestingly, data from previous literature indicated that WNT2B promoted the development of multiple cancers, including NPC (25, 26), cervical cancer (36), ovarian cancer (37), and so on, indicating that WNT2B acted as an oncogene and exerted opposite effects with microRNA-338-5p in regulating cancer progression. Finally, our data showed that the inhibiting effects of microRNA-338-5p overexpression on NPC development were abrogated by upregulating WNT2B, suggesting that microRNA-338-5p targeted WNT2B to suppress cancer progression in NPC.

## Conclusions

Collectively, the present study first identified a novel microRNA-338-5p/WNT2B axis that regulated the development of NPC. Mechanistically, microRNA-338-5p targeted the 3’UTR of WNT2B for degradation, resulting in the inhibiting effects on NPC progression *in vitro* and *in vivo*.

## Data Availability Statement

The original contributions presented in the study are included in the article/**Supplementary Material**. Further inquiries can be directed to the corresponding author.

## Ethics Statement

The studies involving human participants were reviewed and approved by the Ethics Committee of Wuwei People’s Hospital. The patients/participants provided their written informed consent to participate in this study. The animal study was reviewed and approved by the Ethics Committee of Wuwei People’s Hospital.

## Author Contributions

SW: Conception, investigations, resources, manuscript drafting, data collection, and analysis. TY: Technical support, data visualization, and manuscript proofreading. ZH: Conception, guidance, funding acquisition, and manuscript review and submission. All authors contributed to the article and approved the submitted version.

## Funding

This work was financially supported by the Basic Science Foundation of Gansu Province (Grant No. 2016DE56398210), and the funding body supported the present study in terms of resources, data collection/analysis, and manuscript drafting and publication.

## Conflict of Interest

The authors declare that the research was conducted in the absence of any commercial or financial relationships that could be construed as a potential conflict of interest.

## Abbreviations

3’UTRs, 3’ untranslated regions; WNT2B, Wnt family member 2B; NPC, Nasopharyngeal carcinoma; miRNAs, MicroRNAs; circRNAs, Circular RNAs; LncRNAs, Long non-coding RNAs; HIF-1α, Hypoxia-induced factor 1α; ATCC, American Type Culture Collection; FBS, Fetal bovine serum; ECL, Electrochemiluminescence; CCK-8, Cell-counting kit-8; OD, Optical density; FCM, Flow cytometry; PI, Propidium iodide; HNSC, Head and neck squamous cell carcinoma; IHC, Immunohistochemistry.
