# Supplementary material for: Investigations on the Role of the MicroRNA-338-5p/Wnt Family Member 2B (WNT2B) Axis in Regulating the Pathogenesis of Nasopharyngeal Carcinoma (NPC)
Source: Front Oncol. 2021 Jun 29;11:684462. doi: 10.3389/fonc.2021.684462 (PMC8276634; doi:10.3389/fonc.2021.684462)

Figure 3C/N-cadherin

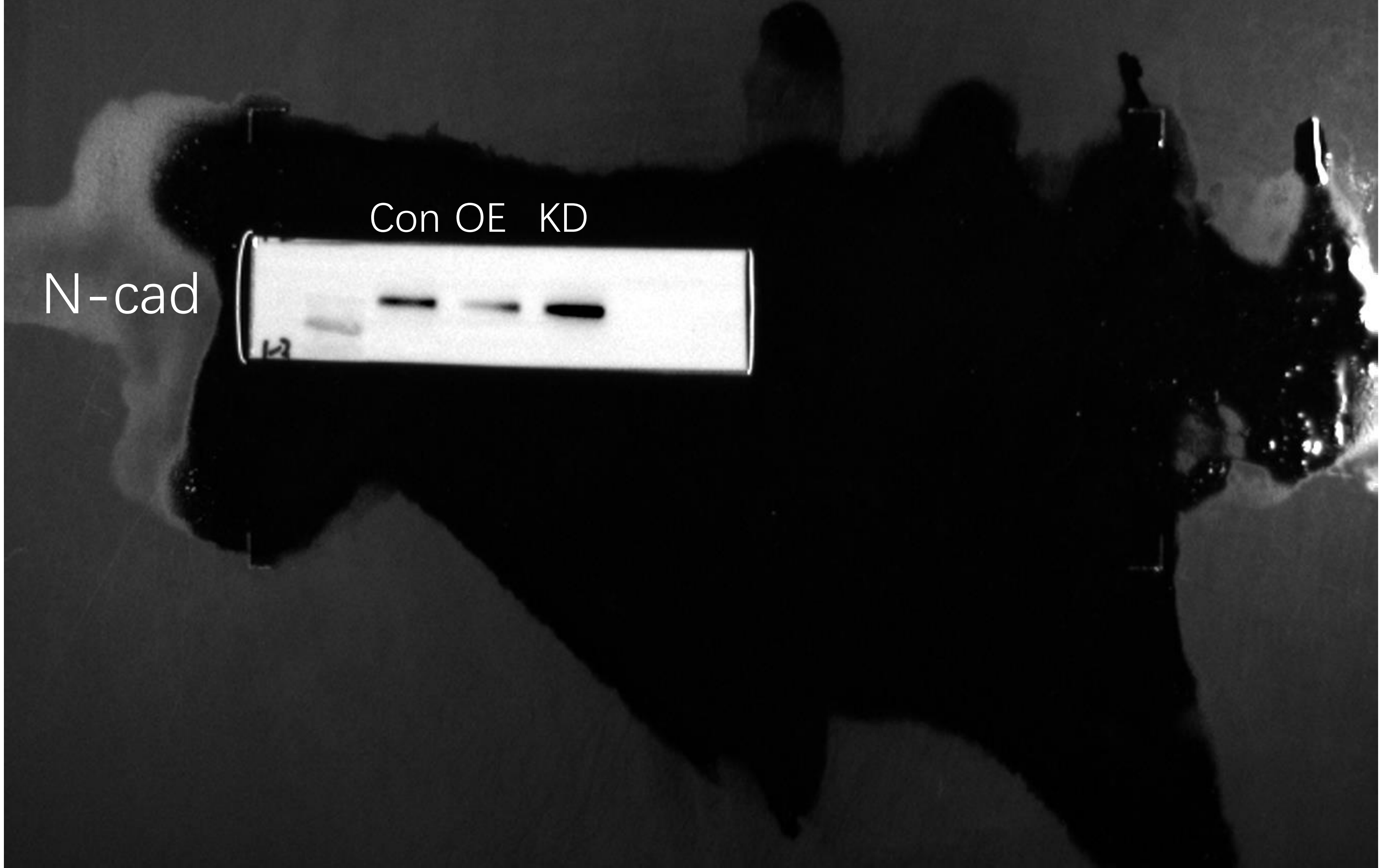

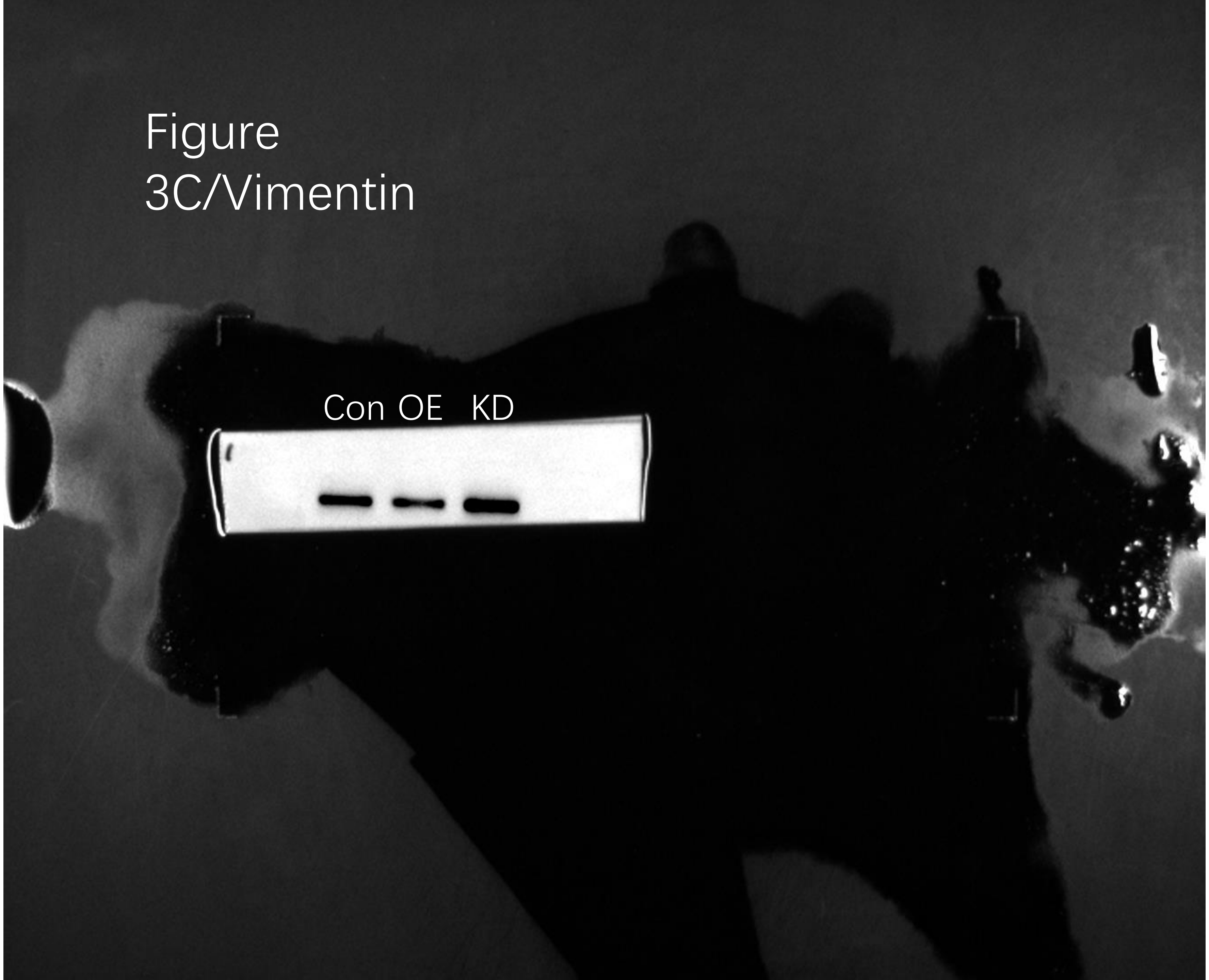
Figure
3C/Vimentin
Con OE KD

Figure 3C/Actin

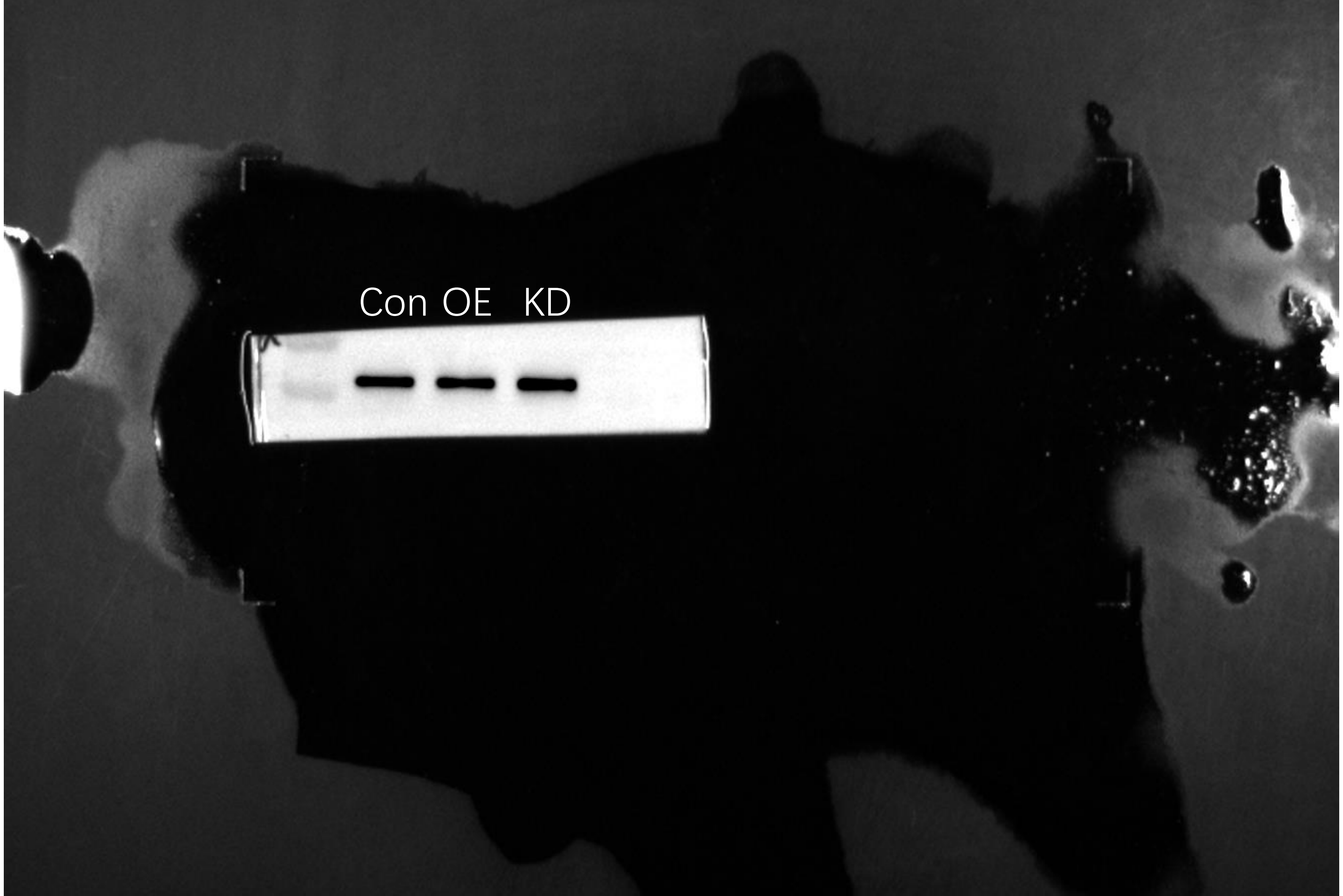

Figure 3D/N-cadherin

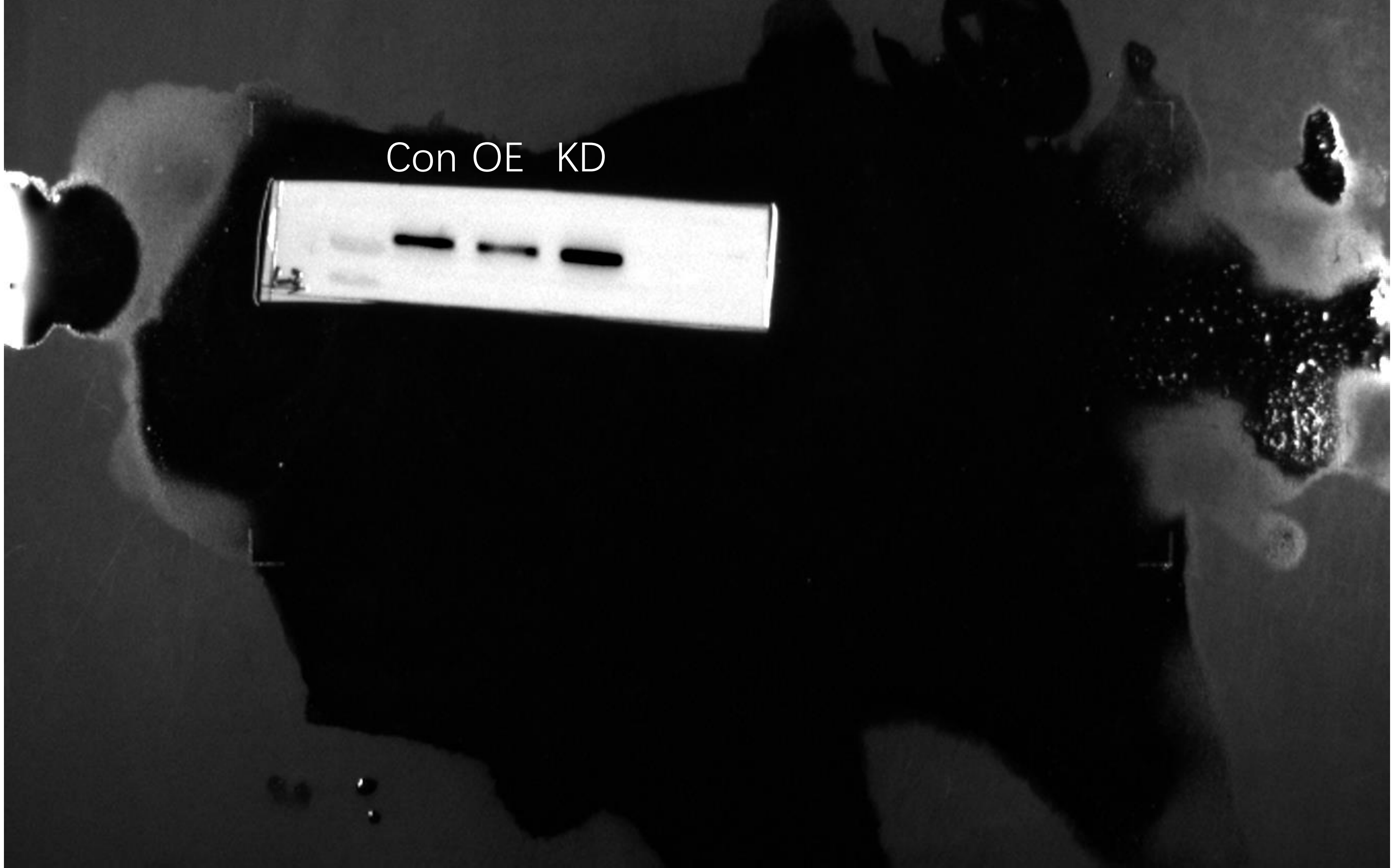

Figure
3D/Vimentin

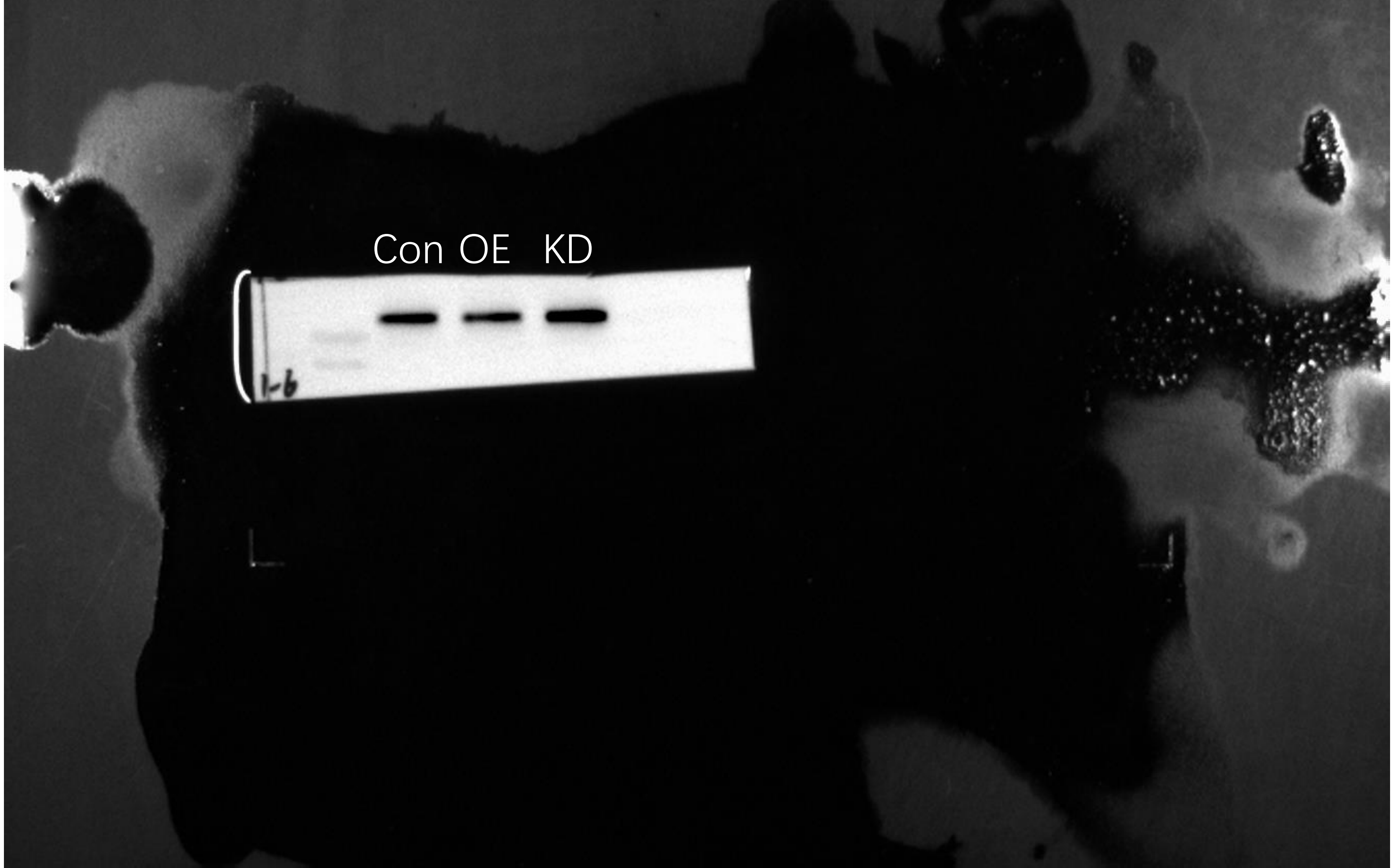

Figure 3D/Actin

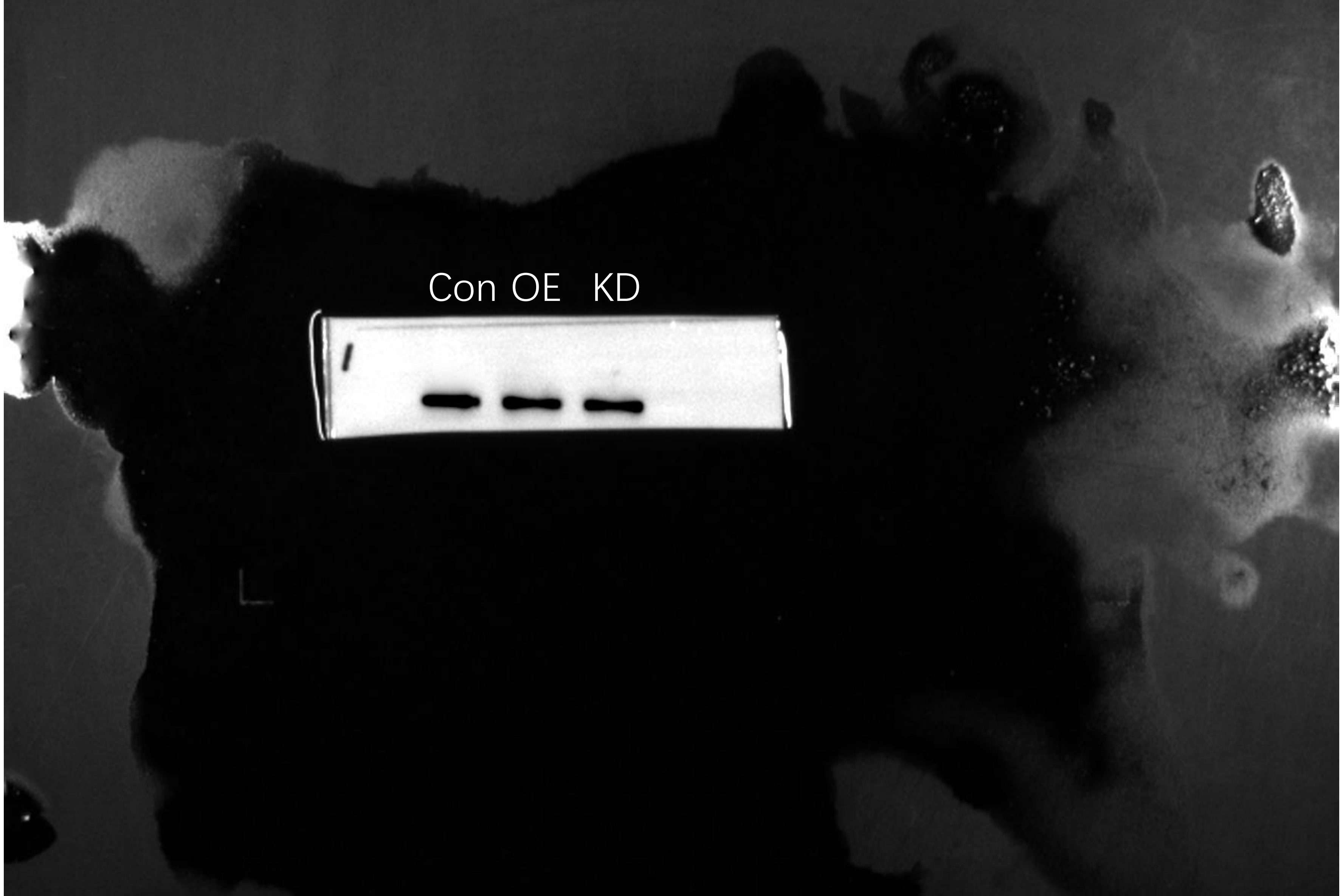

Figure
4E/CNE1/WNT2
B

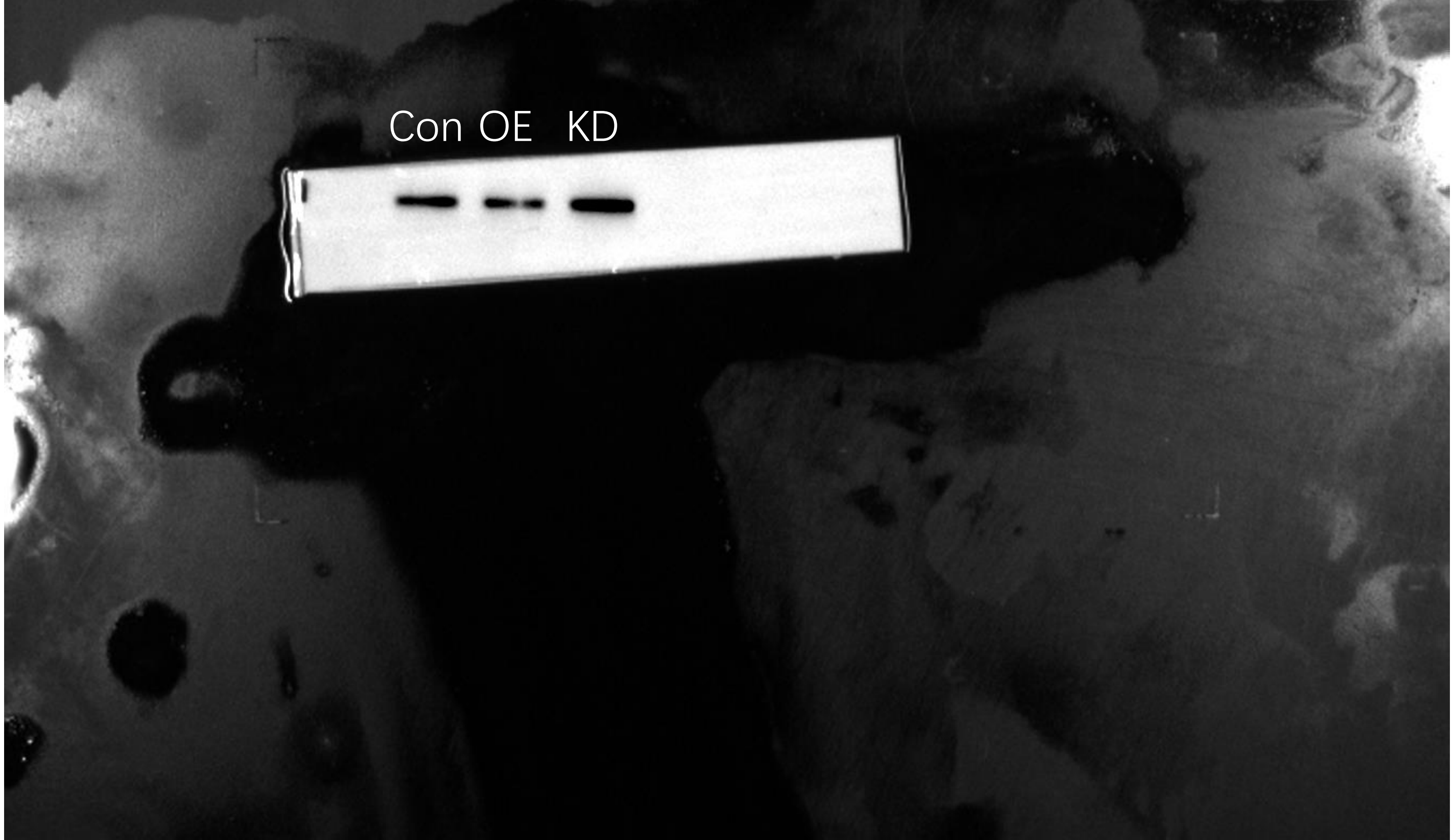

Figure 4E/CNE1/Actin

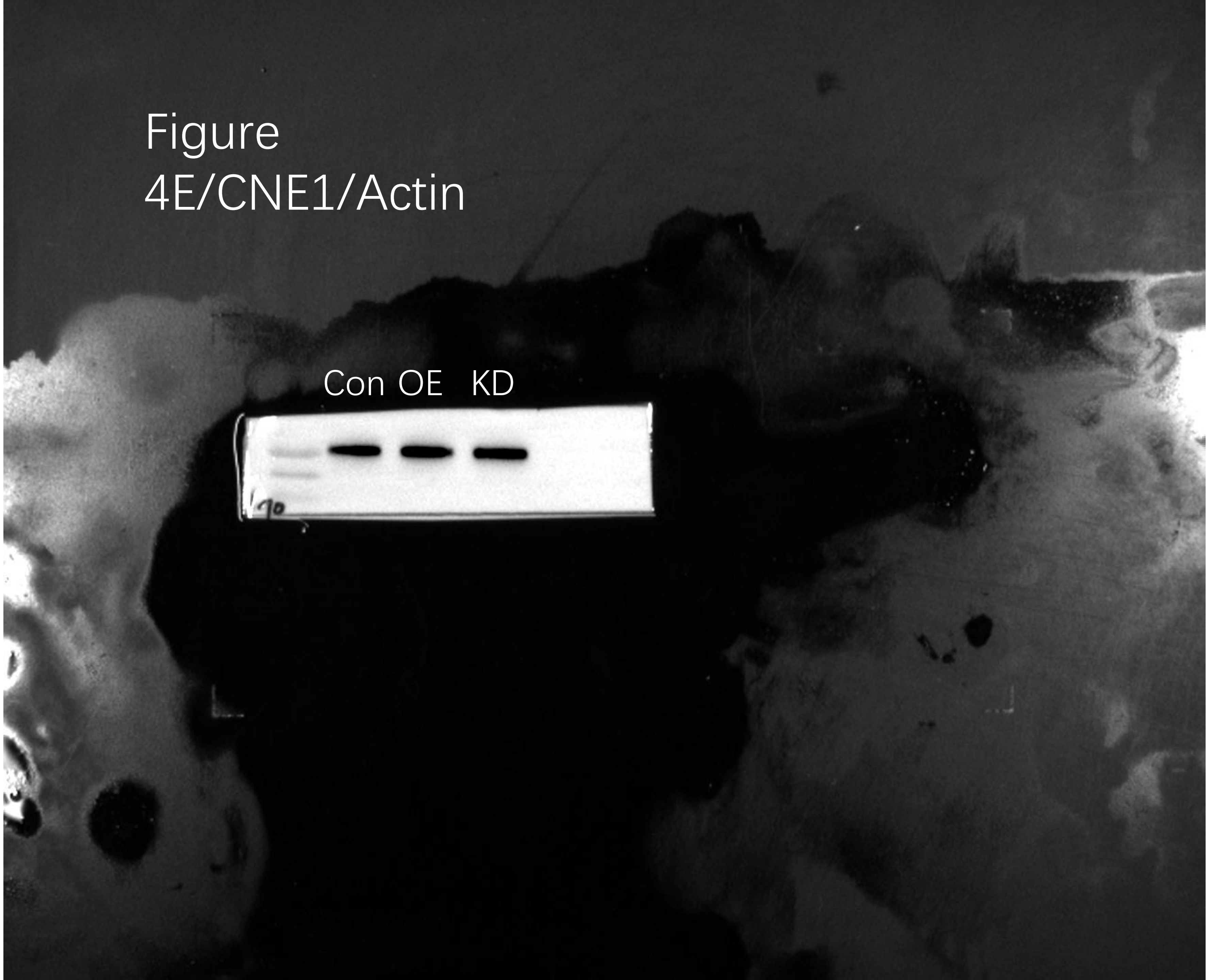

Figure
4E/CNE2/WNT2
B

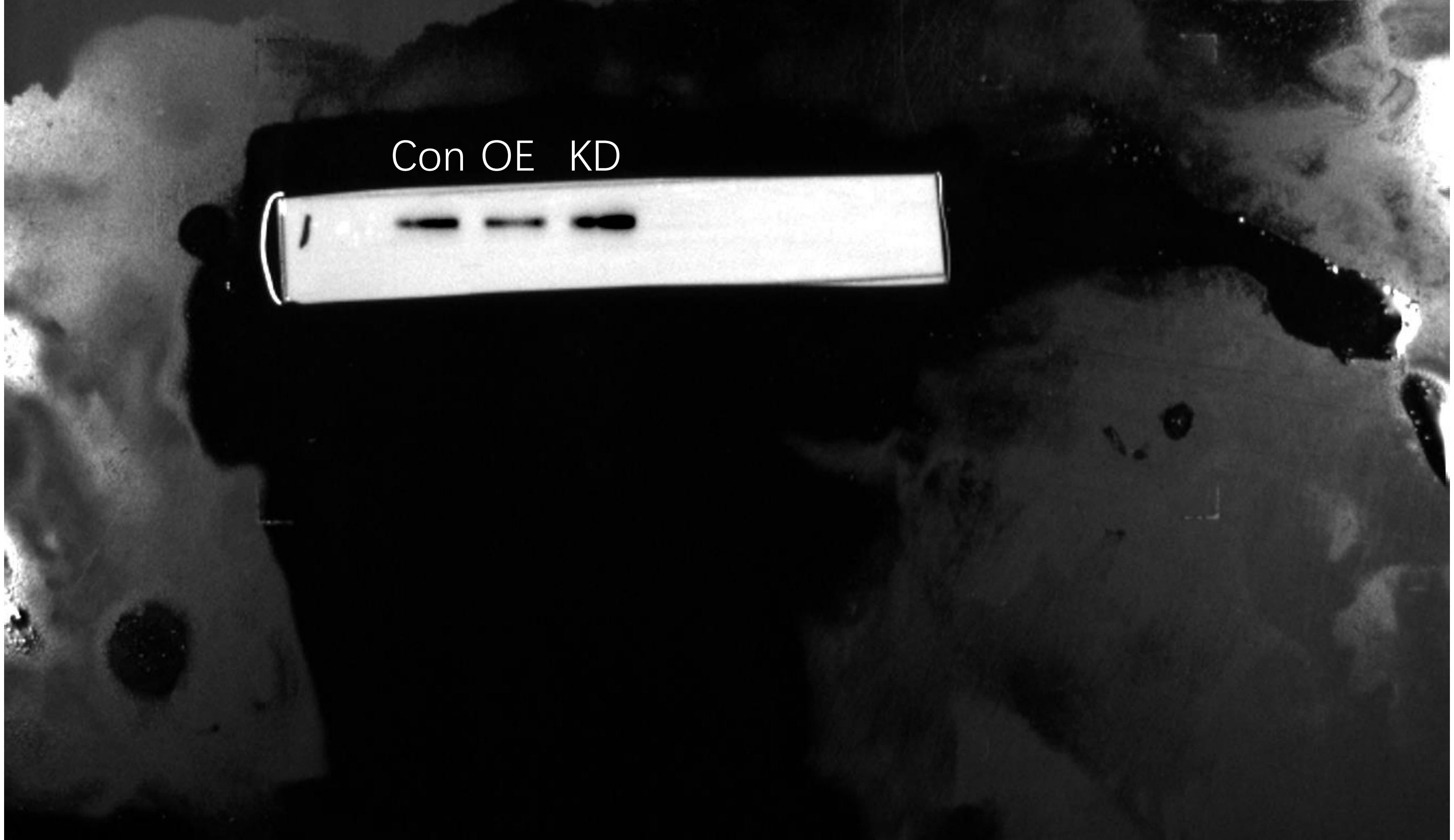

Figure
4E/CNE1/Actin

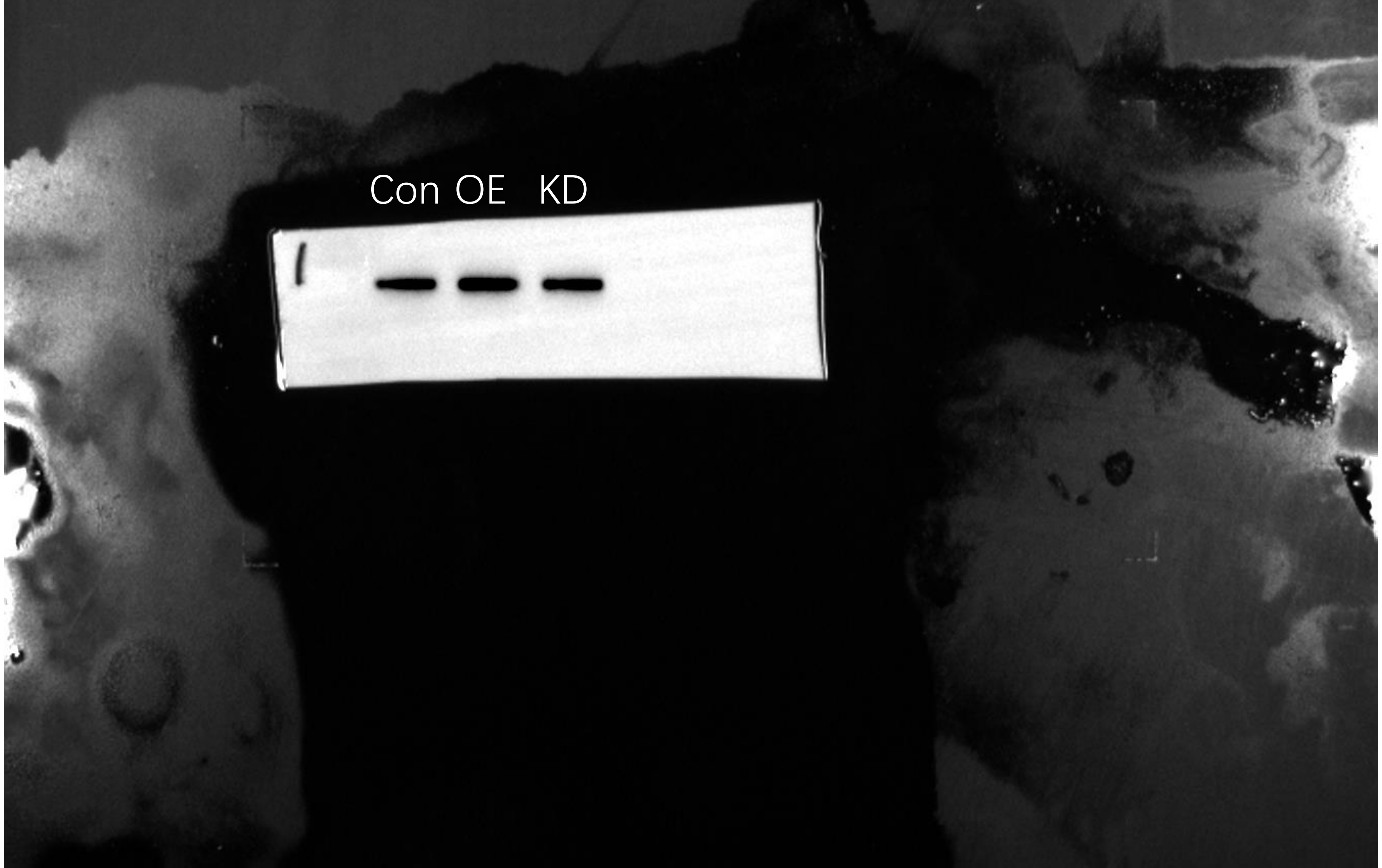

Supplement: Supplementary file 1 [file DataSheet_1.pdf]
