# Supplementary material for: Investigations on the Role of the MicroRNA-338-5p/Wnt Family Member 2B (WNT2B) Axis in Regulating the Pathogenesis of Nasopharyngeal Carcinoma (NPC)
Source: Front Oncol. 2021 Jun 29;11:684462. doi: 10.3389/fonc.2021.684462 (PMC8276634; doi:10.3389/fonc.2021.684462)
Supplement: Supplementary file 2 [file DataSheet_2.docx]

**Supplementary figures and figure legends**


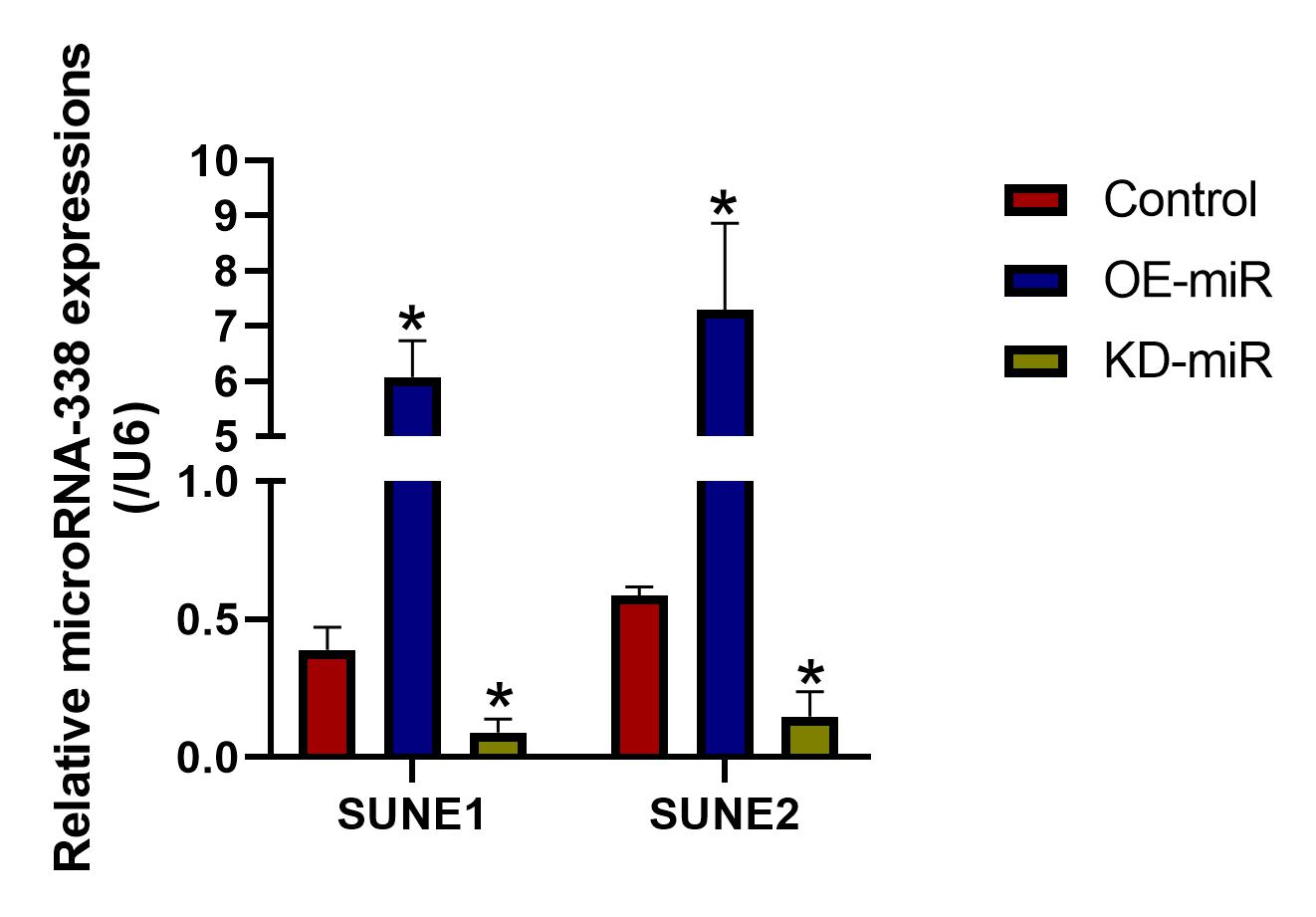


**Figure S1.** MicroRNA-338-5p mimic and inhibitor were successfully delivered into NC cells to overexpress and silence microRNA-338-5p, respectively. Each experiment repeated at least 3 times, and **P* < 0.05.


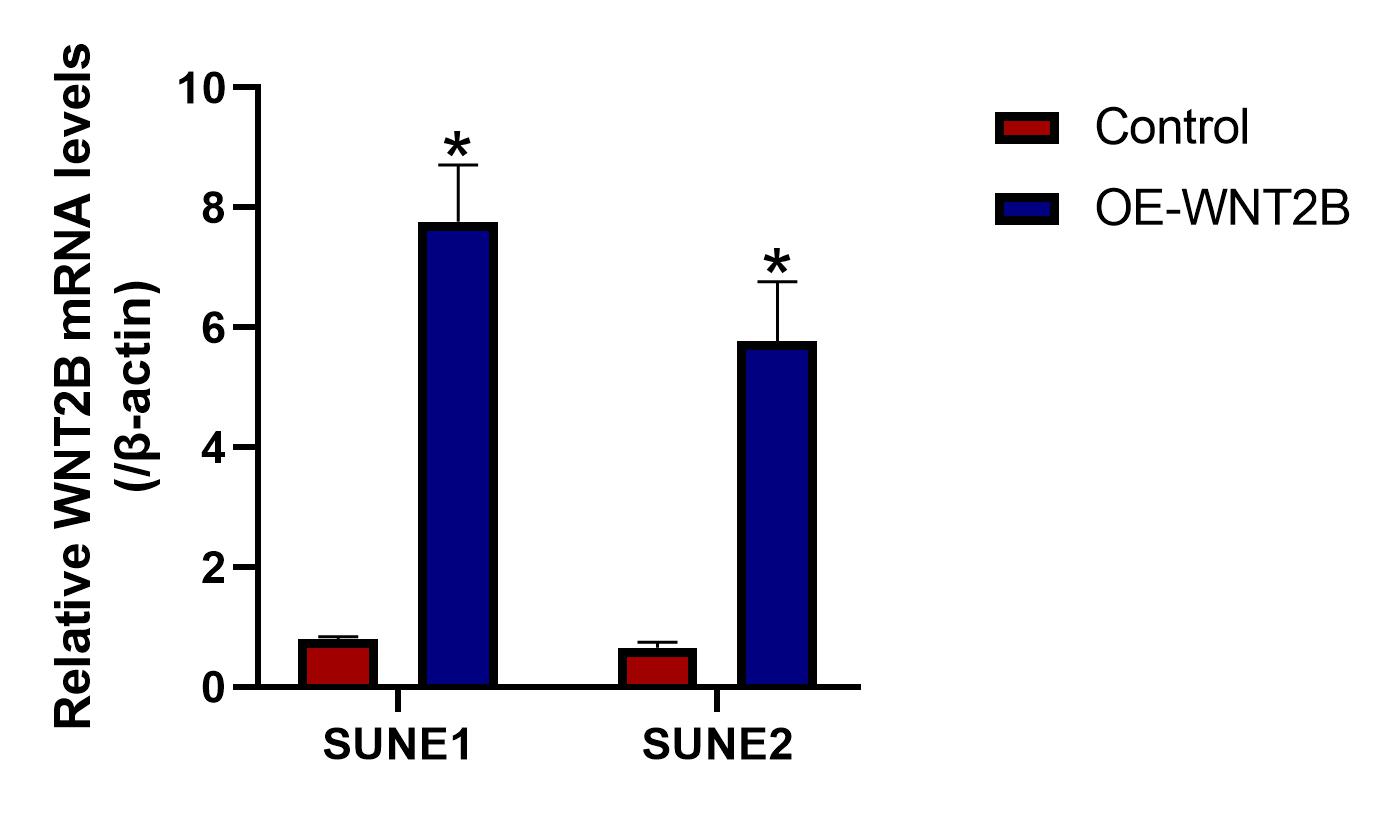


**Figure S2.** The WNT2B overexpression vectors were transfected into NPC cells. Each experiment repeated at least 3 times, and **P* < 0.05.
