# Supplementary material for: Investigations on the Role of the MicroRNA-338-5p/Wnt Family Member 2B (WNT2B) Axis in Regulating the Pathogenesis of Nasopharyngeal Carcinoma (NPC)
Source: Front Oncol. 2021 Jun 29;11:684462. doi: 10.3389/fonc.2021.684462 (PMC8276634; doi:10.3389/fonc.2021.684462)

**Original data for the *in vivo* tumors**

1. SUNE1 cells
   1. Control group


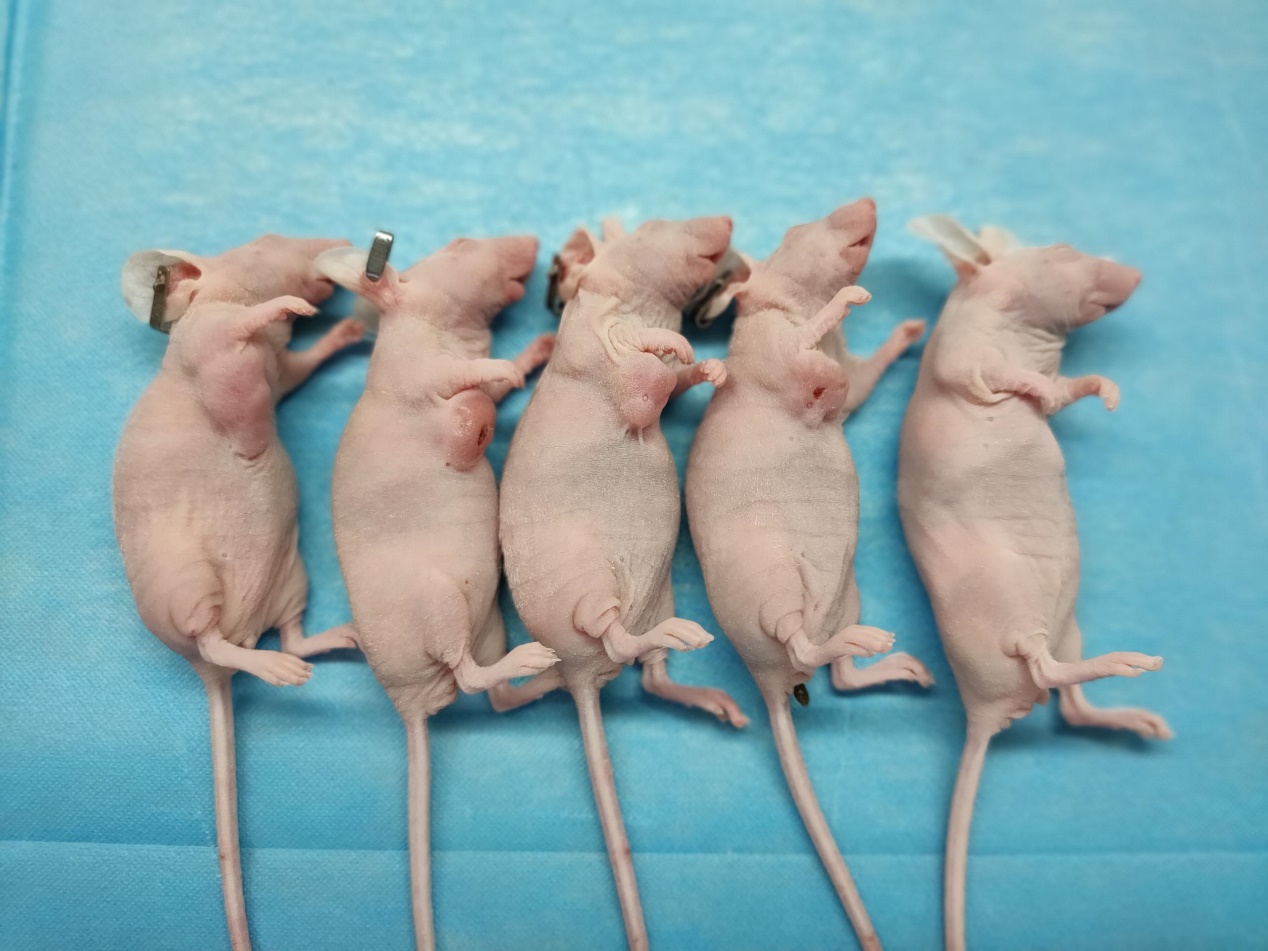


- 1. OE-miR group


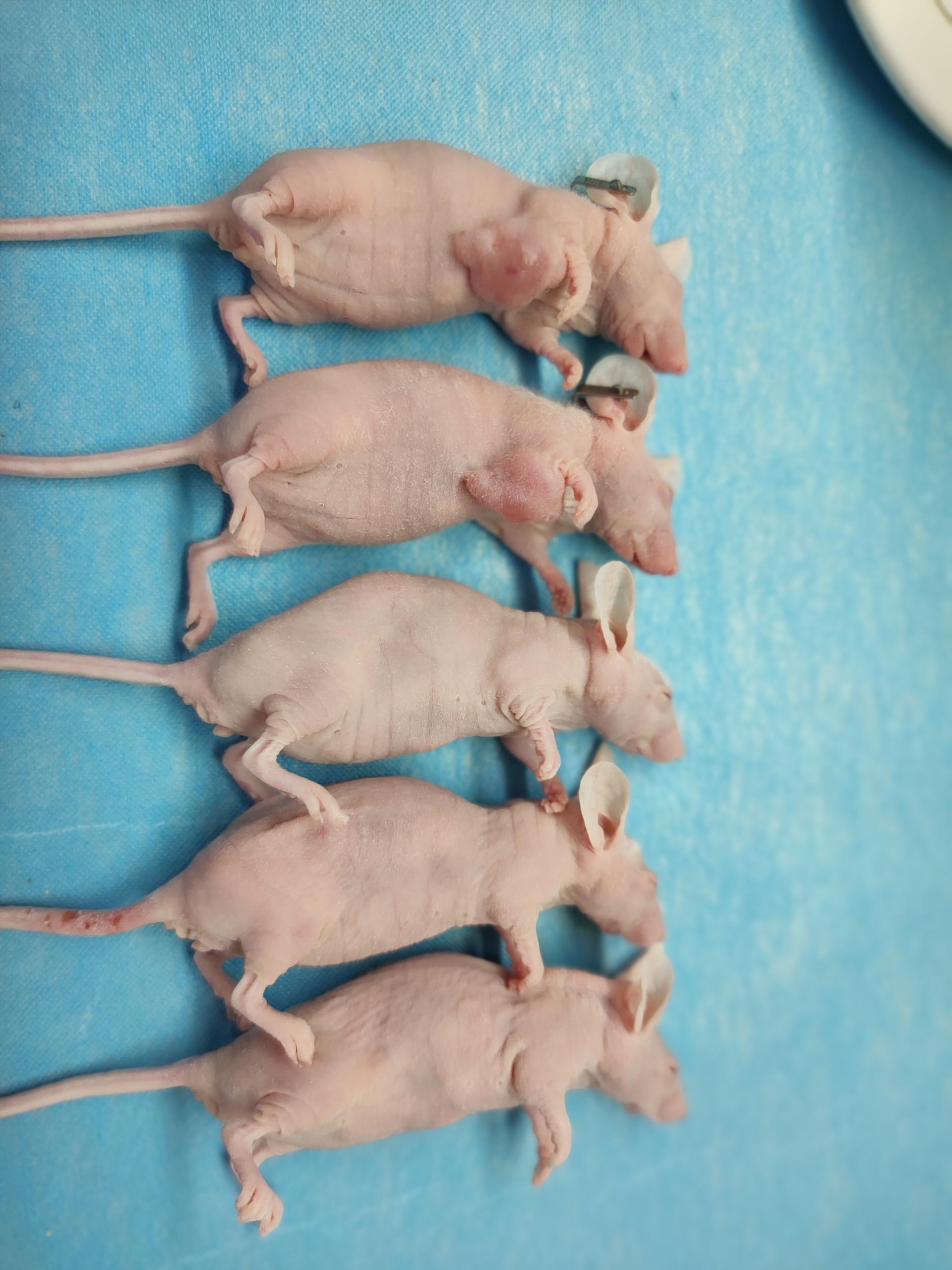


- 1. KD-miR group


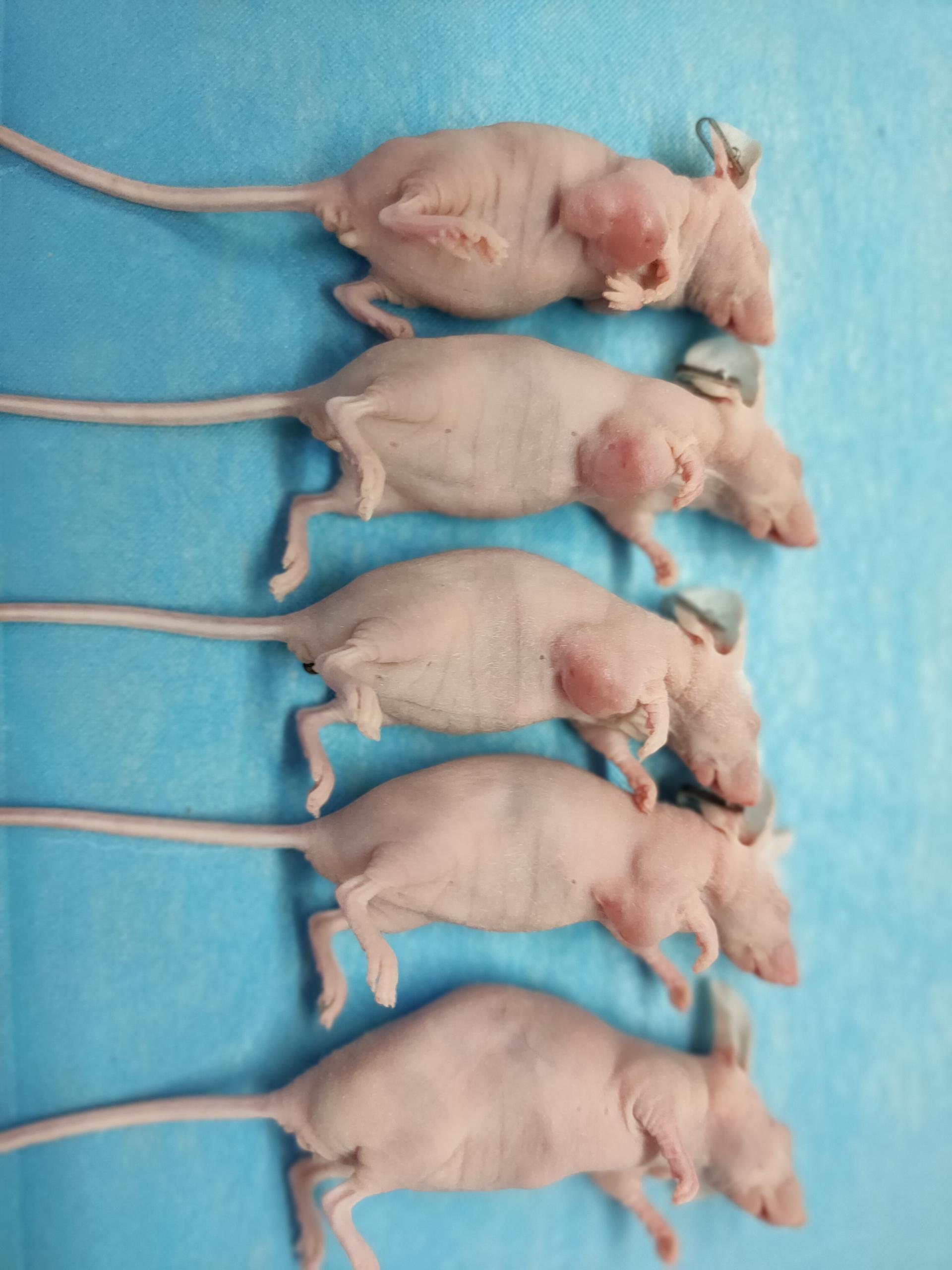


1. SUNE2 cells
   1. Control group


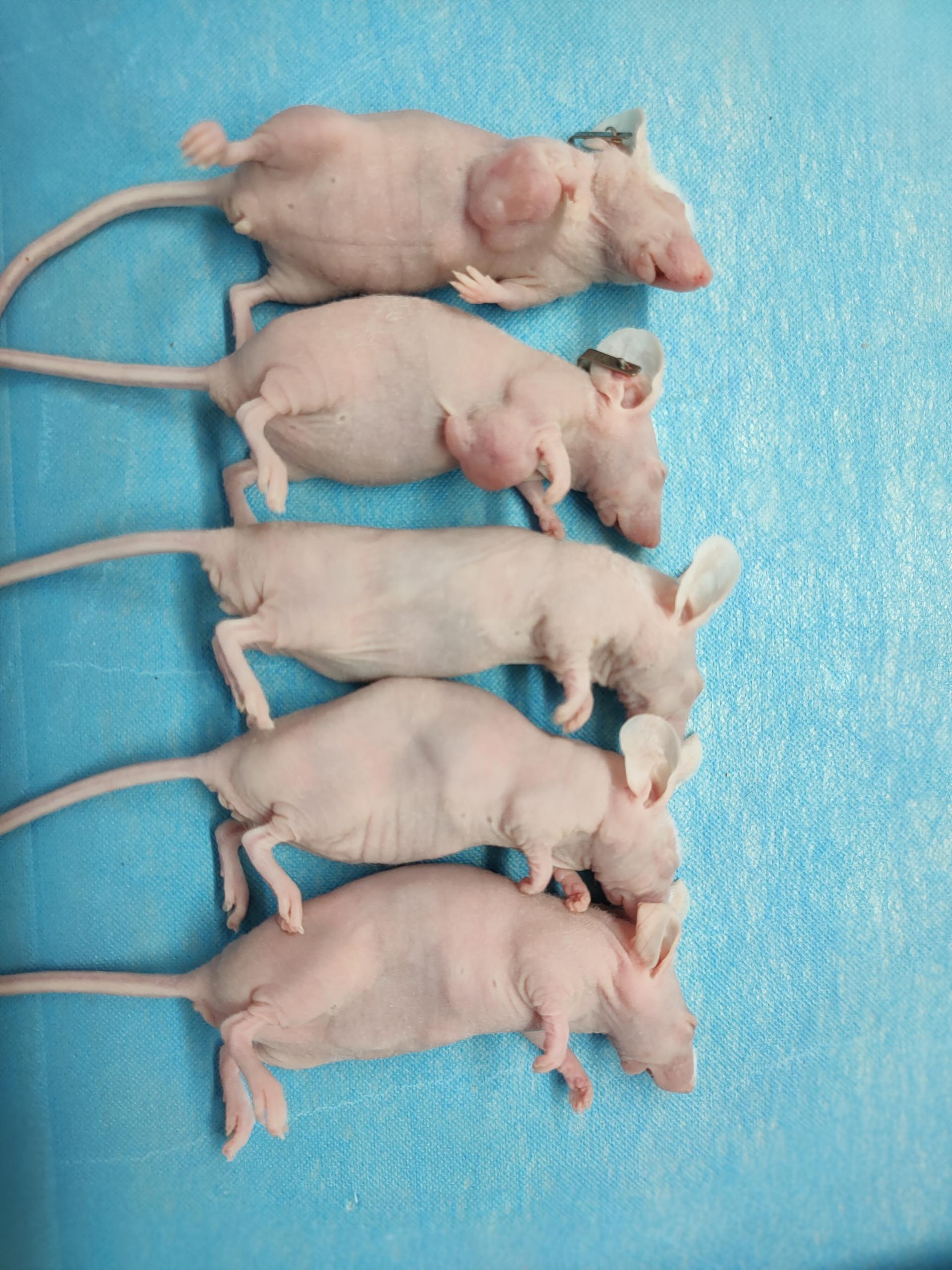


- 1. OE-miR group


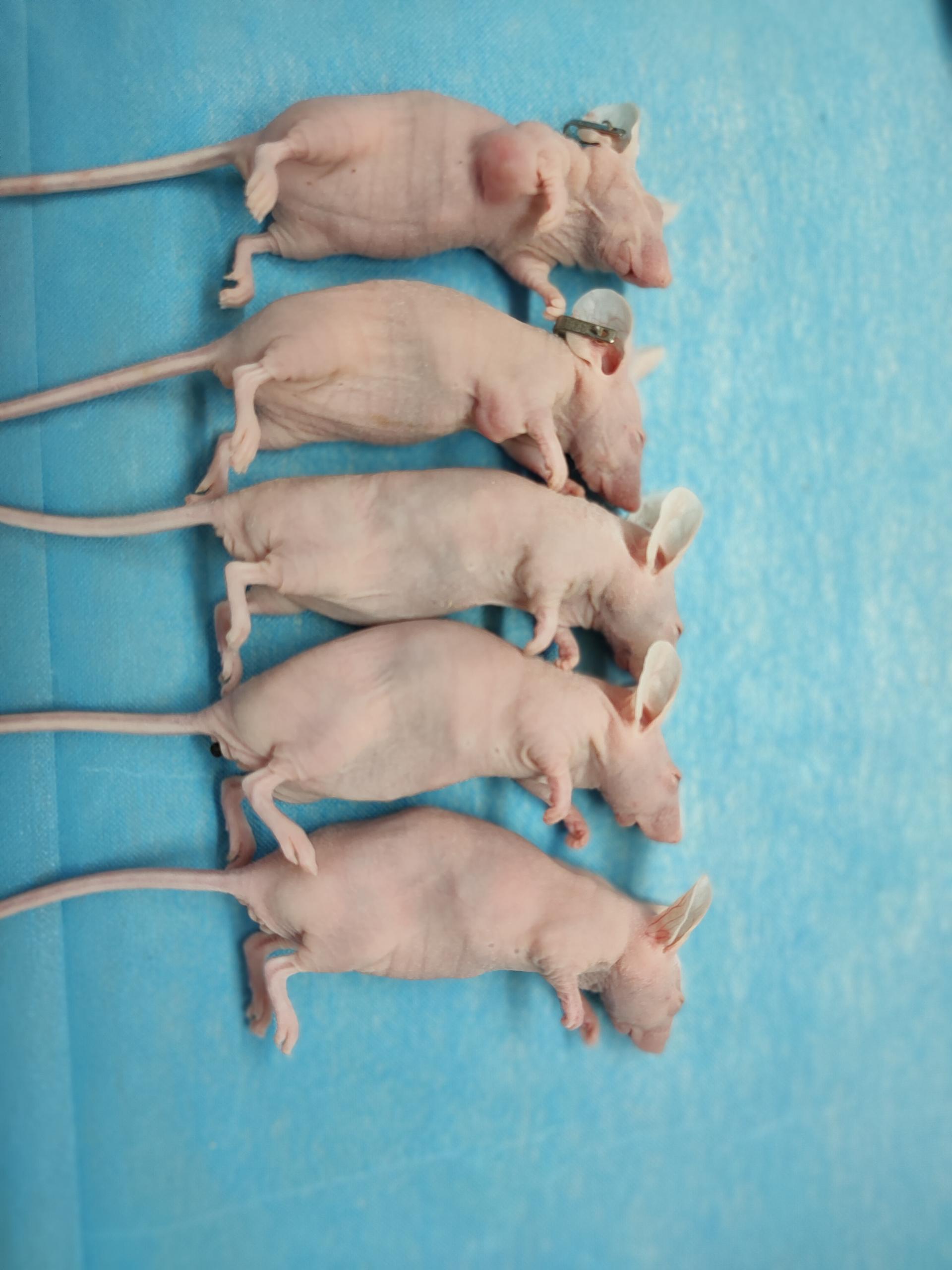


2.3 KD-miR group


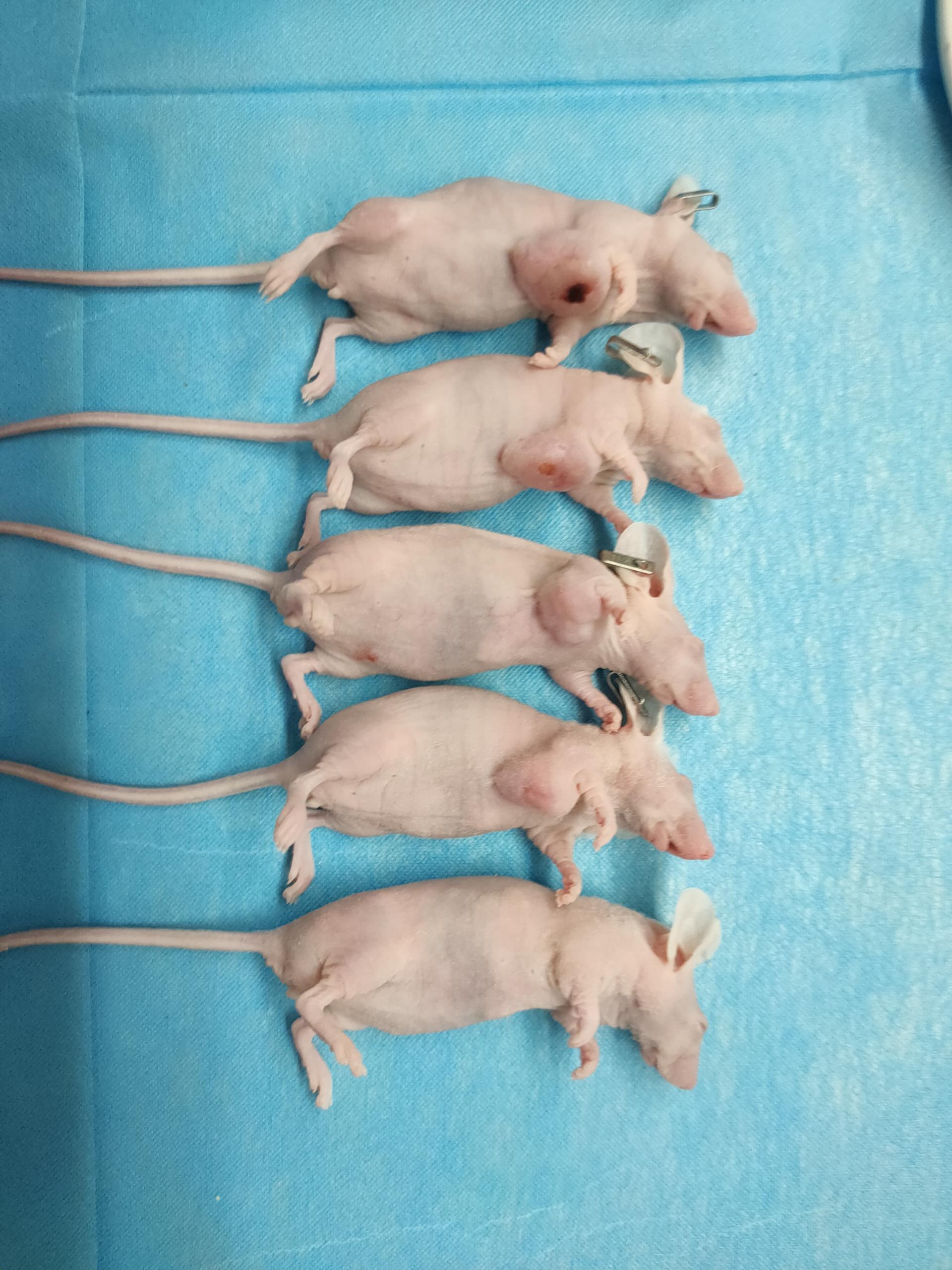

Supplement: Supplementary file 3 [file DataSheet_3.docx]
